# Editorial: Proceedings of IPSC 2019 - 2^nd^ International Plant Spectroscopy Conference

**DOI:** 10.3389/fpls.2020.599481

**Published:** 2020-10-23

**Authors:** Lisbeth G. Thygesen, Andras Gorzsás, Hartwig Schulz

**Affiliations:** ^1^Department of Geosciences and Natural Resource Management, University of Copenhagen, Copenhagen, Denmark; ^2^Department of Chemistry, Umeå University, Umeå, Sweden; ^3^Independent Researcher, Stahnsdorf, Germany

**Keywords:** spectroscopy, microscopy, imaging, plant anatomy, plant physiology, ecology, plant-derived products

The 2nd International Plant Spectroscopy Conference took place in Berlin 2018, where 138 experts from 26 countries gathered to exchange new knowledge on the use of many different types of spectroscopy and microspectroscopy for the study and evaluation of plants and plant products within both academia and industry. The current Research Topic comprises 12 highlights from the conference, illustrating the broadness of the field, expanding into chemometrics.

The main part of the contributions illustrates how different types of spectroscopy can be used to explore plant tissues and how they develop with the aim of understanding the numerous ways in which physiology and anatomy are linked. Xiao et al. used X-ray tomography, infrared spectroscopy and Raman imaging to follow the maturation of walnut shells, and found that cell wall thickening and lignification was followed by an accumulation of fluorescent compounds. Piqueras et al. also studied the development of a specialized plant tissue, namely heartwood in a larch species, in this case by use of synchrotron infrared microspectroscopy and atomic force microscope infrared spectroscopy. They found that the heartwood formation observed is consistent with a process where phenolic precursors to extractives accumulate in the sapwood rays, and subsequently are oxidized and/or condensed in the transition zone and spread to the neighboring tracheids in the heartwood. Cuello et al. used infrared imaging to study the cell wall chemistry of different cell types in poplar tension wood, and confirmed that cellulose is abundant in the so-called G-layer of the xylem cells within this tissue, but also found differences within the S-layer of the cell walls. Kendel and Zimmermann used Raman and infrared spectroscopy to compare the chemistry of pollen from more than 200 plant species showing the different strengths of the techniques and demonstrating that they are ideally best used together when studying pollen. In particular, FT-Raman spectra were found to be strongly biased toward the chemical composition of pollen wall constituents, while FTIR over-represented chemical constituents of the grain interior. In the study by Füchtner et al., gas chromatography coupled with mass spectroscopy was employed to study heartwood extractives of a spruce and a larch species with regard to their wood protection properties toward a brown rot fungus. The two tree species were found to rely on two different defense strategies.

Another group of contributions used spectroscopy in studies with an ecological focus, i.e., interactions between plants and their environment. The study by Karimi et al. showed that near infrared spectroscopy coupled with gas chromatography/flame ionization and gas chromatography/mass spectrometry could classify *Zataria multiflora* plants into chemotypes, and that these types could be linked to growth conditions. Diehn et al. used infrared spectroscopy, Raman spectroscopy, surface enhanced Raman scattering, and matrix-assisted laser desorption/ionization mass spectrometry to study pollen samples collected from populations of the grass *Poa alpine* in three different European countries. The molecular information from the four types of spectroscopy was combined with phenotypical and environmental information of the parent plants and populations. Correlations were generally not strong, but it was found that the mass spectroscopy method could differentiate between pollen from the three populations, while the three other techniques could separate between pollen produced under different growth conditions. In the study by Staková et al., identification and quantification of (groups of) peatland plant species by use of infrared spectroscopy of plant roots was tested with some success.

A third group of studies concerned the use of spectroscopy for quality control of plant-derived products. The study by Yan et al. discussed the use of hand-held spectrometers within herbal medicine, and presented two examples, where a hand-held device based on near infrared spectroscopy was successfully used to differentiate similar species. The study also illustrates that a reliable calibration model is pivotal to such applications. In another study, still within herbal medicine, Brangule et al. successfully tested the use of two different variants of infrared spectroscopy for species identification of herbal products and for separation of leaves from flowers. Still within this group of studies, Kanski et al. used gas chromatography based methods to evaluate the quality of tomatoes after two different storage regimes.

The last contribution included in this Research Topic is an up-to-date review by Bec et al. on the use of spectroscopic imaging for the study of plants.

The wide span of the topics included in the present compilation demonstrates the versatility of different types of spectroscopy both within plant sciences and for fast, non-destructive quality control of plant products, especially when combined with chemometrics. The data analysis approaches used in the studies varied with the spectroscopic techniques and the purpose of the studies, and covered a wide span of multivariate techniques. Naturally, principal component analysis (PCA), the “workhorse” of chemometrics, features heavily, along with classical statistical tools (e.g., *t*-tests). However, with more directional analyses and especially with combined datasets (from e.g., multimodal approaches), more guided and specialized techniques were skillfully applied, from analysis of variance (ANOVA) to multivariate curve resolution (MCR), the latter being especially useful for improving interpretability and performing dimension reduction in less abstract terms than PCA (working with spectral and concentration profiles instead of scores and loadings).

The works of this special issue highlighted that no single technique (e.g., vibrational spectroscopic, mass spectrometric, or chromatographic data) can be considered the ultimate tool. Each of them has specific advantages and disadvantages (e.g., the versatility of vibrational spectroscopy vs. the specificity of mass spectroscopy vs. the resolution and speed of fluorescence imaging, etc.), and the key for research groups working at the intersection of plant sciences and spectroscopy is to have a versatile toolbox, where each tool has a well-defined purpose and application area in which it excels, and is complemented by other tools in other areas. The challenge (especially for principal investigators) in this respect is to know the tools well enough to select the optimal one for each task, to be able to coordinate a multitool approach and last but not least, to effectively combine the results with scientific rigor and statistical accuracy. As the contributions show, the biannual International Plant Spectroscopy Conference series provides an excellent forum to strengthen skills along these lines, as it promotes the exchange of knowledge and ideas.

## Author Contributions

All authors listed have made a substantial, direct and intellectual contribution to the work, and approved it for publication.

## Conflict of Interest

The authors declare that the research was conducted in the absence of any commercial or financial relationships that could be construed as a potential conflict of interest.

